# Anxiety and Fear Avoidance Beliefs and Behavior May Be Significant Risk Factors for Chronic Opioid Analgesic Therapy Reliance for Patients with Chronic Pain—Results from a Preliminary Study

**DOI:** 10.1093/pm/pnab069

**Published:** 2021-02-17

**Authors:** Marcelina Jasmine Silva, Zhanette Coffee, Chong Ho Yu, Marc O Martel

**Affiliations:** 1 Founder and former Medical Director of The Focus on Opioid Transitions Program, Integrated Pain Management Medical Group Inc, Walnut Creek, California, USA; 2 College of Nursing, University of Arizona, Tucson, Arizona, USA; 3 Department of Behavioral and Applied Science, Azusa Pacific University, Azusa, California, USA; 4 Faculty of Dentistry & Department of Anesthesiology, McGill University, Quebec, Canada

## Abstract

**Objective:**

To describe differences between patients with chronic, non-cancer pain (CNCP) who were successfully able to cease full mu agonist chronic opioid analgesic therapy (COAT), and those who exhibited refractory COAT reliance, among those who participated in a multidisciplinary program designed for COAT cessation.

**Design:**

A retrospective review of electronic medical records (EMR) data was organized for preliminary analysis.

**Setting:**

A multicenter private practice specializing in CNCP, which received patient referrals from the surrounding geographical area of primary and specialty care offices in Northern California.

**Subjects:**

Data from 109 patients with CNCP who participated in a multidisciplinary program to cease COAT between the dates of October 2017 to December 2019 were examined.

**Methods:**

EMR data, pre-COAT cessation, of oral morphine milligram equivalence (MME) and validated questionnaire responses assessing anxiety and fear-based beliefs and behavior, as well as opioid misuse, were extracted and compared between those who successfully ceased COAT and those who did not.

**Results:**

Patients who were unsuccessful at COAT cessation reported significantly higher Fear Avoidance Beliefs Questionnaire (FAB) scores. No significant differences were found based on incoming MME amounts, Current Opioid Misuse Measure (COMM) or Tampa Scale of Kinesiophobia (TSK) scores. Pain Catastrophizing Scale (PCS) scores showed a split pattern with unclear significance.

**Conclusions:**

Results suggest that fear avoidance beliefs and behavior, as measured by the FAB, play a significant role in refractory COAT reliance for patients with CNCP.

## Introduction

### The Negative Sequelae of Chronic Opioid Analgesic Therapy (COAT)

The practice of using full mu agonist chronic opioid analgesic therapy (COAT) in the setting of CNCP has had detrimental impacts on an individual as well as a societal level in The United States [1–3]. Even when used as prescribed, COAT use is associated with significant negative clinical outcomes for the individual patient. Long-term side effects include hypogonadism [4] and immunocompromise [5]. A myriad of adverse immediate effects are also common [6], such as constipation, dry mouth, urinary retention, emotional blunting and cognitive impairment. COAT can also lead to overdose-related mortality, even in patients who use it exactly as prescribed, in times of physiological stress or unfortunate medication interaction. On the societal level, COAT is a significant contributor to the concerningly high levels of mortality and morbidity imparted by the opioid epidemic—due to compliant use as well as diverted prescription drug misuse—which has decreased the average American lifespan in recent years compared to other developed countries [1, 3, 7].

In terms of a comprehensive treatment approach, the practice of using COAT for CNCP has been correlated to worsened outcomes. COAT has been shown to impede vocational and social return to function and increase length of disability in injured workers [8]. It is also associated with increased systemic inefficiencies and healthcare utilization as well as medical-legal actions [9]. In one study of over 1,300 participants with chronic disabling occupational spinal disorders, patients dependent upon opioids had a significantly greater length of disability (24.54 vs 17.08 months), were 2.5 times as likely to have had surgery and were 1.5 times more likely to be represented by an attorney when compared to case controlled patients with similar pathology who were not dependent upon opioids [9].

Called to action by the 2016 guidelines from The Centers for Disease Control and Prevention [1], the medical community has been discussing best-practice approaches to decrease the use of COAT for CNCP. Differing approaches have been met with varying levels of success of opiate cessation [10–40]. Despite these efforts, a definitive best-practice treatment approach to the conundrum of COAT reliance in CNCP remains elusive. This may be related to the fact that still little is known about the variables that influence refractory COAT reliance in the first place.

### Anxiety and Fear-Based Beliefs and Behavior

Anxiety and fear-based beliefs and behavior have been strongly implicated in certain aspects of the negative chronicity experienced by those who suffer from CNCP. Such beliefs and behavior have been associated with increased disability [19, 43–48] , pain intensity, emotional distress [43], and absenteeism [19]. Studies have shown, and replicated, that fear of movement and reinjury is a better predictor of self-reported disability than biomedical findings or pain intensity levels [49, 50] . Anxiety and fear-based beliefs and behavior have also been documented to affect opioid use in terms of prolonging postoperative use, increasing opioid craving, and contributing to general misuse [51–54].These behavioral trends drew researchers for this present study to question whether COAT reliance in CNCP is similarly sustained by anxiety and fear-based beliefs and behavior.

Clinically similar schematics between COAT reliance and the negative chronicity of CNCP further support the hypothesis that both phenomena have a foundation in anxiety and fear-based beliefs and behavior. First, fear avoidance of pain, from the stance of learning theory, is a self-perpetuating dynamic in which anticipated consequences require little reinforcement to create long-term habitual behavior [55].Expectations of pain hinder physical activity, regardless of actual pain, and this expectation is rarely confronted, so is not disproved, leading to deconditioning and further disability [44, 56, 57].This self-perpetuating, learned dynamic is also applicable in the context of COAT, as many patients associate the action of taking a scheduled opioid with that of prophylactically avoiding or escaping pain, and thus rarely confront the unadulterated experience of their physical pain, spiraling deeper into the habit and resulting sequalae of COAT use. This dynamic is even more entrenched in the case of opiate use, as it is reinforced by dopaminergic incentivization and abrupt abstinence syndrome disincentivation [58]. Second, fear-based avoidance of physical activity may be initially adaptive, but becomes maladaptive when applied chronically, as it leads to deconditioning, further injury, increased pain, social withdrawal, and even depression [59]. Similarly, opioid therapy is initially adaptive in the contexts of acute injury and peri-operative pain management, but the sequalae of COAT inflict those who do not cease use. Third, despite the fact that many experience an acute low back pain episode at least once during a lifetime, only a small minority develop a chronic low back pain problem [60]. Likewise, many people may utilize a short course of opiate analgesics for an acute injury, but do not go on to require COAT, regardless of the severity of injury.

The Fear Avoidance Beliefs Questionnaire (FAB), Pain Catastrophizing Scale (PCS), and Tampa Scale for Kinesiophobia (TSK) are frequently employed to assess anxiety and fear-based belief and behavior. While these tools assess similar phenomena, their interchangeability has been negated in investigative comparisons [50, 61–64], posing the possibility that they each may have specific applications within the realm of anxiety and fear-based beliefs and behavior assessment. Some have also been proposed to be pragmatic tools to investigate and improve treatment model efficacy. Targeted psychosocial therapy to improve PCS scores has been shown to be efficacious in expediting return to work after a period of disability [61]. FAB analysis may help determine which clinical interventions will have an increased probability of a successful outcome for patients with CNCP [44, 65, 66].

### The Fear Avoidance Model

**Figure 1:**
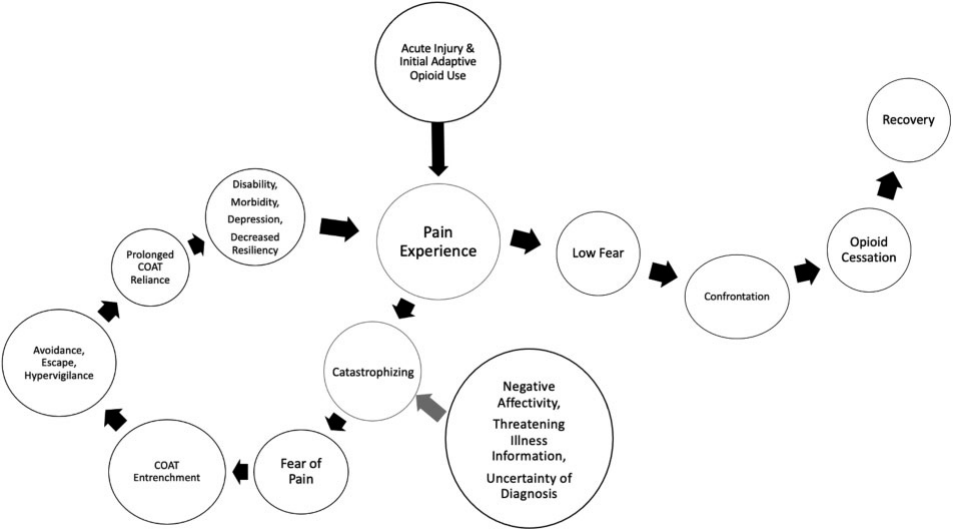
Fear avoidance visual model of COAT reliance. (Adapted from Leeuw [69], Lethem [67], Vlaeyen [68], and Waddell [44].)

There are so many similarities between the classic fear avoidance model of CNCP [44, 67,68–69] and the clinical course of COAT, that the fear avoidance model can be coopted to illustrate the different paths for patients who rely upon COAT versus those who don’t (Figure 1). The basic tenet of the model is that the way in which pain is interpreted leads to two potential pathways. When pain is perceived as no or low threat, patients are likely to only use a short course of opiates. In contrast, a vicious circle may be initiated when the pain is catastrophically misinterpreted, giving rise to pain-related fear, and associated avoidance/escape (opioid use) and hypervigilance. In both models, uncertainty about a diagnosis leads to increased fear avoidance beliefs, regardless of the pathological severity or anatomical patterns of pain [44]. While the avoidance route can be adaptive in the acute pain stage, it paradoxically entrenches and strengthens the reliance upon COAT in the subacute and chronic stages of pain, which invites negative chronicity. Eventually, the long-term consequences, such as disability [8] and morbidity [1, 3] and depression [51, 70–73], further decrease the ability to access resilience-building, non-COAT pain coping mechanisms.

### Study Objectives

The above similarities prompted an exploration of anxiety and fear-based beliefs and behavior in relation to COAT use in the clinical setting. The inquiry undertaken by the researchers was: if it is so that disability and negative chronicity in the setting of CNCP is strongly motivated by anxiety and fear-based beliefs and behavior [44, 47], could it also be that COAT reliance in the setting of CNCP is similarly motivated? To further that inquiry, the present study was undertaken with the objective to examine the variables associated with COAT cessation outcomes among patients with CNCP who participated in a multidisciplinary program designed for COAT cessation.

## Methods

### Study Design

Data were collected via a retrospective review of the electronic medical records (EMR)—decoupled from individually identifying features––comparing the incoming measures of 109 patients with CNCP who participated in a multidisciplinary program [73] designed to promote COAT cessation. Standardized and previously validated psychological questionnaires were given to each patient at orientation (pretreatment and pre-COAT cessation). Comparisons were made between the pretreatment questionnaire scores, incoming MME, and gender of those who successfully ceased COAT use and those who did not. Data were analyzed for participants in the programs that ran between the dates of October 2017 to December 2019. Measures from every patient who started the program were included in the study. As measures were taken from the first day of the program, no data was lost from program incompletion. Those who did not graduate from, or complete, the program or who failed to cease COAT were considered unsuccessful and were included in the data as such. Unsuccessful patients have been labeled as being refractorily reliant upon COAT for the purposes of this paper.

The multidisciplinary program commenced as a circumscribed, higher acuity treatment entity under the auspices of a multicenter private practice specializing in CNCP, which received patient referrals from the surrounding geographical area of primary and specialty care offices in Northern California. These referral sources were diverse and included Federally Qualified Health Care Centers, unaffiliated private practices, hospital-affiliated clinics, occupational medicine providers and workers compensation entities. Two centers and two different clinical teams participated in administering the program. Funding for the program occurred via routine medical fee-for-service billing for treated patients. The researchers analyzed the data presented here through the course of program-related quality assurance measures. No other funding was present for this study. This study was reviewed by a private IRB and was determined exempt from full review.

### Participants

By default, the subjects of the present study met the inclusion criteria of the clinical program—adults who consented (as opposed to were mandated) to a group program for the purpose of COAT cessation and carried a diagnosis of CNCP from any etiology; had used daily COAT at the time of admission or had struggled to maintain recent opioid cessation; had tried and failed or plateaued on an opioid wean previously; and were failing to meet realistic functional and analgesic goals, despite participation in traditional outpatient COAT for CNCP treatment, as determined by the individual patients and/or their primary care teams. COAT used by participants at program initiation were any form of commercially available oral or transdermal long and short-acting preparations obtained while under the care of a physician. Program participation was determined via a semi-structured, motivational interviewing style evaluation between each participant and one of the qualified program clinicians.

Exclusion criteria from the program were candidates who:

- Did not carry a diagnosis of CNCP
- Were actively engaging in opioid diversion
- Had an active substance use disorder, or comorbidity, of significant acuity to be appropriate for higher than Level 1 (outpatient) services as defined by The American Society of Addiction Medicine (ASAM) [77] , including: a biomedical or psychological comorbidity of moderate intensity or higher (ASAM Dimensions 2 and 3, respectively; Level 2 or higher intensity) [77] during the proposed time of program participation.
- Had a neurocognitive or neurodegenerative disorder that precluded the ability to actively participate in care-planning decisions and/or reliably follow written instructions.

### Intervention

The group multidisciplinary program was built around a standardized curriculum designed to transition patients with CNCP off COAT. The treatment occurred once a week in a 6-hour session for 10 weeks. The curriculum entailed group cognitive behavioral therapy (CBT) emphasizing pain coping skills and mood regulation, complementary care modalities delivered in a group setting (such as biofeedback, mindfulness, acupuncture and gentle motion), and individualized medication management. Every activity was led by a licensed or credentialed expert in that field—such as a physician, nurse practitioner, psychologist, licensed acupuncturist, and physical therapy assistant. Extended panel urine drug screen was mandated at every meeting to corroborate participant compliance. Participants were determined to have successfully graduated from the program if COAT cessation was achieved in one of four ways: a complete transition to buprenorphine, an abstention without transitional medication assistance, a maintenance of recent COAT abstinence in the context of reported struggle with craving or coping, or a reduction of incoming buprenorphine dose by over 50% in incoming patients not using full mu agonist opioids for analgesia. For the purposes of this paper, graduation and COAT cessation, as defined by the pathways above, are synonymous.

### Measures

#### Pain Catastrophizing Scale

The PCS determines patients' levels of pain catastrophizing [43]. Catastrophic thinking has been implicated as a risk factor for increased disability length, pain intensity and emotional distress, as well as prolonged time out of work after a physical injury [43]. Several studies have supported the reliability and the validity of the PCS among patients with chronic pain [46, 51, 53, 69]. A score of 30 or more has been declared clinically relevant [43], however, lower scores have been associated with chronicity of prolonged recovery and delayed return to work [62].

#### Fear Avoidance Beliefs Questionnaire—Work and Physical Activity

The FAB consists of two subscales: The Work subscale (FAB-W) and The Physical Activity subscale (FAB-PA). Several studies have supported the validity and reliability of the FAB for the assessment of fear avoidance among patients with CNCP [49, 68, 69, 75, 79]. The optimal cut off for determining a significant FAB score in relation to negative chronicity in CNCP has been studied in several contexts and varies accordingly [44, 68, 75, 77–80]. A higher FAB score been shown to correlate with an increased probability of current and future work loss and disability [19, 44, 47] as well as social withdrawal [48]. Of note, some of the utility of the FAB-W has been shown to differ between privately and industrially insured patients [66].

#### Tampa Scale of Kinesiophobia

The TSK is a measure of fear of movement, injury or reinjury. It is reliable with a Cronbach alpha of 0.77 [49]. A score of 37 or over is considered a score for clinically significant Kinesiophobia [49], though different score percentiles have been validated for backpain versus fibromyalgia [49, 81, 82]. TSK scoring is interconnected with decreased physical performance and increased pain intensity, depressive symptoms, pain‐related anxiety, and perceived disability [45].

#### Current Opioid Misuse Measure

The COMM is a self-report questionnaire that was developed to identify patients prescribed COAT for CNCP who are exhibiting aberrant and/or opioid misuse behavior [83]. The COMM was designed to help clinicians stratify levels of monitoring or specialty referrals for patients with CNCP using COAT. A score of 9 or greater identifies a patient who is at high risk of opioid misuse or abuse with a 77% sensitivity and 66% specificity [83]. The COMM screens for problematic behavior that may increase opioid misuse risk but does not differentiate between the causes of behavior (i.e., mood disorders, general non-adherence, addiction, etc.), and thus, positive scores may warrant different clinical responses [84].

### Data Analysis

#### Fisher’s Exact Test and Probability Plot

The Fisher’s exact test [85–87] was conducted to determine whether incoming MME (Table 1) was related to COAT cessation, as it amends the potentially invalidating issue of a low cell count of categorical variables, when compared to χ^2^ statistics. Originally, incoming MME was a continuously-scaled variable; however, the distribution was skewed. To rectify the situation, incoming MME was converted into an ordinal variable using the value of 90 as the cutoff; 90 MME was chosen because it is the generally recognized cutoff for high dose opiates, as the 2016 CDC guidelines suggest clinicians should avoid increasing dosage beyond it [1].

**Table 1.** Incoming MME by ultimate outcome of unsuccessful vs successful COAT cessation

CountCol %Row %	Unsuccessful	Successful	Total
Low (90 MME or less)	9	75	84
	81.82	76.53	
	10.71	89.29	
High (> 90 MME)	2	23	25
	18.18	23.47	
	8.00	92.00	
Total	11	98	109

COAT = chronic opioid analgesic therapy; MME = morphine milligram equivalence.

#### Penalized Regression

Penalized regression, also known as generalized regression [88], was utilized to identify the potential predictors of the failed COAT cessation based on psychological questionnaires (Table 2). In traditional ordinal logistic regression, the variable selection process is subject to the order of entering the potential predictors. Thus, usually there is no unique solution and the model may be unstable across different samples. In penalized regression when different predictors enter the model, a penalty is imposed on the model in order to avoid complexity. There are different types of penalized regression and in this study elastic net, which integrates LASSO and ridge regression, was chosen. In LASSO the regression coefficients of unimportant variables were assigned as zero while in ridge regression the regression coefficients of those predictors were shrank towards zero. The term “elastic net” is so named because different paths to the solution were explored in order to identify the best model.

**Table 2.** Psychological assessment indicators of unsuccessful coat cessation via the penalized regression model

Variable	Estimate	Std. Error	Wald χ^2^	*P*-value	Lower 95% CI	Upper 95% CI
Pretesting: PCS	0.8043529	0.0945233	72.41278	<0.0001*	0.6190906	0.9896152
Pretesting: FAB-PA	7.2716044	0.9384139	60.044282	<0.0001*	5.432347	9.1108618
Pretesting: FAB-W	−1.545901	0.2034496	57.736388	<0.0001*	−1.944655	−1.147147
Pretesting: COMM	0	0	0	1.0000	0	0
Pretesting: TSK	0	0	0	1.0000	0	0

COMM = Current Opioid Misuse Measure; FAB-PA = Fear Avoidance Behavior Physical Activity; FAB-W = Fear Avoidance Behavior Work; PCS = Pain Catastrophizing Scale; TSK = Tampa Scale of Kinesiophobia.

#### Linking and Brushing

**Figure 2:**
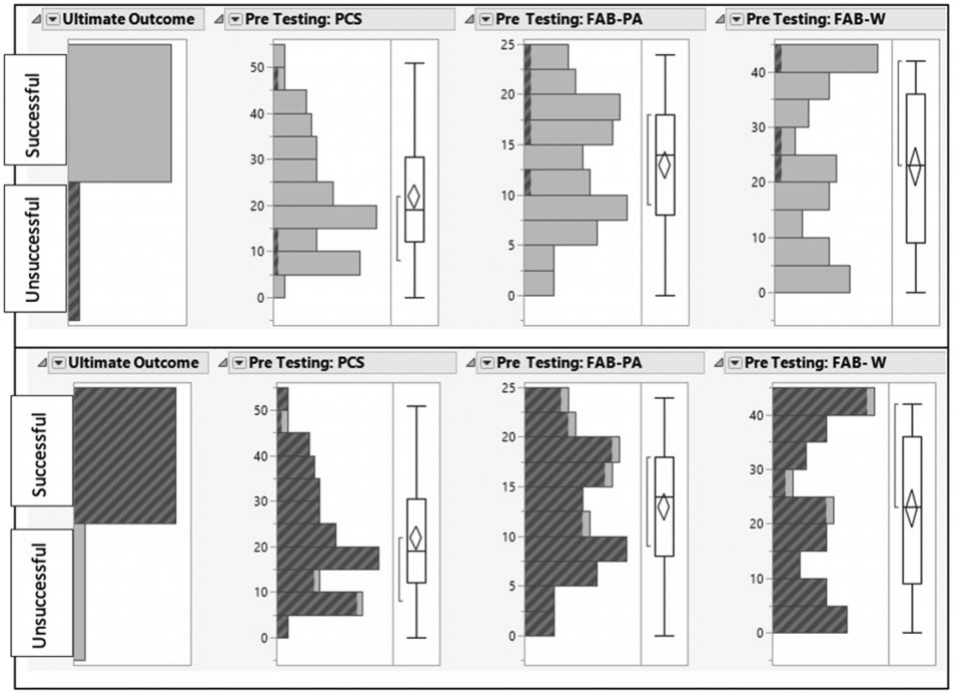
Linking and brushing between COAT cessation and psychological assessment questionnaire scores.

After significant predictors of the outcome were identified by penalized regression analysis, linking and brushing (Figure 2) [89], which is a data visualization technique, was employed to examine the relationships between the outcome and the predictors. In this dynamic visualization approach, the distributions of the dependent and independent variables are displayed in inter-connected panels. When certain observations are shaded in one panel, the corresponding observations are also shaded so that the inter-relationships between variables can be revealed.

#### Decision Tree

**Figure 3:**
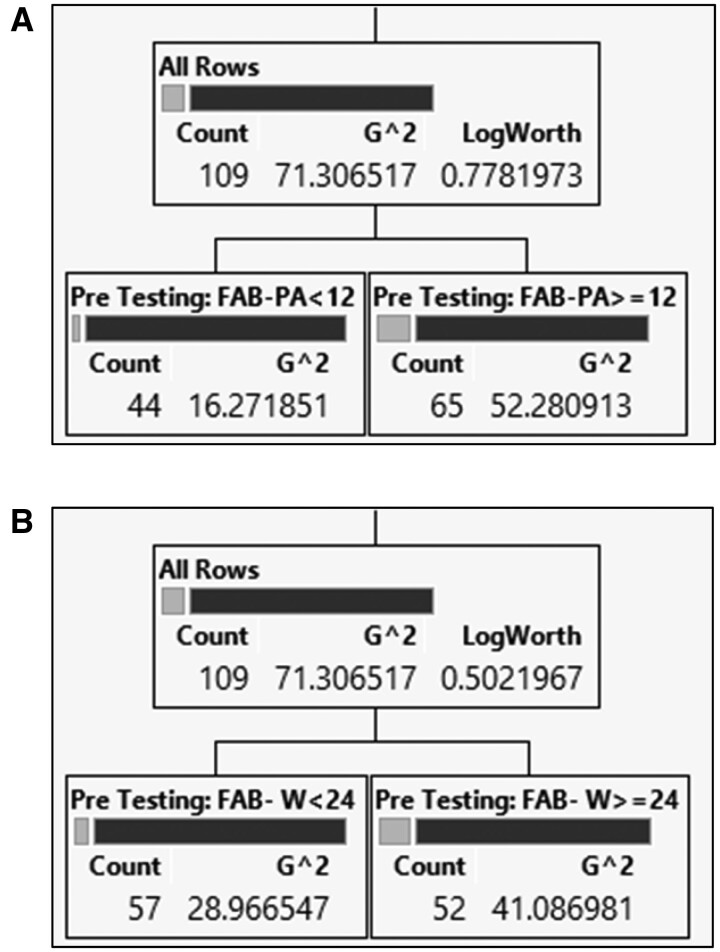
(A., B) Decision tree of FAB score cut off with reference to unsuccessful COAT cessation. (**A, B)**, Light gray bar represents the observations with unsuccessful COAT cessation, whereas the black bar denotes those with successful COAT cessation. According to the left node of the decision trees, when FAB-PA is less than 12, and the FAB-W is less than 24, only a few participants are unsuccessful.

The decision tree approach, also known as the partition tree or the classification tree [90, 91] was employed to determine the threshold of FAB-PA and FAB-W scores that conferred risk of failed opiate cessation (Figure 3A and B). The partition process in the decision tree is built upon information theory with the goal of achieving homogeneity of the partitioned group. In the process the decisive split-point was found so that observations that are similar in terms of the dependent outcome can be grouped together.

## Results

### Descriptive Statistics and Gross Observations

Descriptive statistics were derived from the retrospective data found in the demographic and prescription sections of the EMR. A 90% success rate of participant COAT cessation by the time of graduation was revealed across all participants. Participants ranged in age from 27 to 88 years old; 69% were identified as female. There were only two unsuccessful male participants, making the association between gender and COAT cessation indeterminant, due to the small sample size.

Inferences can be made about the socioeconomics of the studied population based on the program design and the EMR report of patients’ insurance payers: approximately 30% of participants were insured by Medicare, 25% by industrial insurers, 10% by Medicaid, <1% participated without insurance, and the remainder had commercial insurance (approximately 44%). Program participation occurred during customary business hours, making it only available to participants who had flexible work schedules, were not working due to retirement or disability, or were employed part-time.

### MME via Fisher’s Exact Test

Table 1 is the crosstab of COAT cessation success by incoming MME. Fisher’s exact test amends the issue of low cell counts and indicates a nonsignificant *P* value: .7069.

### Psychological Assessments Scores via Penalized Regression

The result of penalized regression, as shown in Table 2, reveal that the scores of PCS, FAB-PA, and FAB-W were found to be significant indicators of unsuccessful COAT cessation. The regression coefficients of COMM and TSK were shrank to zero, meaning that these two variables were not significant.

### Linking and Brushing between Outcomes

The relationship between the outcome and the predictors were further investigated by linking and brushing. The top panel of Figure 2 indicates that all patients who were unsuccessful to cease COAT have high FAB-PA and FAB-W pretest scores. However, in the same observations the PCS pretest scores were both high and low. Hence, while there is an association between high FAB-PA&W scores and unsuccessful COAT cessation, the relationship between PCS and lack of success is not clear. The bottom panel of Figure 2 shows that there is no discernable pattern among patients who were successfully able to cease COAT in relation to the significant predictors. To be specific, this regression model has no predictive power for successful COAT cessation, only potentially for refractory COAT reliance.

### FAB Threshold via Decision Tree

The decision tree approach, also known as the partition tree or the classification tree [90, 91] was employed to determine the decisive split-point, or cut off, for FAB scores that correlate with unsuccessful COAT cessation. In Figure 3A and B, the grey bar represents the observations with unsuccessful COAT cessation, whereas the black bar denotes those with successful COAT cessation. According to the left node of the decision trees, when FAB-PA is less than 12, and the FAB-W is less than 24, only a few participants are unsuccessful. It is crucial to emphasize that the threshold for risk of unsuccessful COAT cessation was determined by the algorithm, not the analyst.

## Discussion

The present findings give strength the theory that elevated anxiety and fear-based beliefs and behavior support COAT reliance, much as they support disability and the negative chronicity of CNCP [69], as measured by the FAB. The consistently elevated FAB-PA score for patients unable to cease COAT use suggests that fear avoidance of physical activity is a significant factor for refractory COAT reliance. Though the FAB-PA value of 12––suggested here as a threshold for risk––is low, relative to previous studies [44, 77], it is consistent with the phenomena that differing FAB scores have been reported to be significant amongst different populations for differing purposes [75, 77, 80] .These findings suggest a potentially novel application for the FAB-PA, which has historically been less well correlated with specific outcomes when compared to the FAB-W [44, 75, 77]. The present results for the FAB-W hold up less well to external validation, as this questionnaire was originally validated for patients who are currently, or were recently, working [44, 75, 77]. and validity was shown to change based on insurance payer type [66, 78]. As the current study did not separate participants who identified as disabled or retired, nor did it control for insurance type, the significance of the FAB-W results is uncertain in this study.

It is notable that the PCS and TSK did not uniformly trend with the FAB-PA in relation to refractory COAT reliance. The consideration must be entertained that the FAB-PA results could be a red herring and should be confirmed with repeated or larger studies. However, while the TSK, FAB, and PCS all measure anxiety and fear-based beliefs and behavior around pain, previous investigations into their instrumental interchangeability have frequently failed to show cross-over reliability without reducing contributory revelations [50, 61–64]. The present study is consistent with previous studies that lacked interchangeability, suggesting that COAT cessation may be an additional area in which applicability of these assessment instruments differs. This phenomenon supports previous calls for the research and development of more specific assessment tools for targeted biopsychosocial screening [68], which could potentially be used to direct more efficient risk identification tools and interventions for refractory COAT reliance.

Also remarkable in this study is the data pertaining to COMM scores, in that there was no correlation between higher scores and refractory COAT cessation. It has been warned in previous COMM validations that this test is vulnerable to respondent manipulation and false report [92], which could affect the generalizability of our findings. However, the potential that refractory COAT reliance in the setting of CNCP is more strongly correlated to anxiety and fear-based beliefs and behavior than to aberrant opioid-related behavior, would unveil nuances underlying this reliance, which is frequently misunderstood as a phenomenon bordering opiate use disorder as defined by the DSM-5 [93]. The current findings may help better define and distinguish different motivations that manifest in similar displays of opioid reliance, thus setting the stage for more refined avenues of assessment and treatment for similarly presenting opioid-related behaviors.

Finally, the noncontribution of incoming COAT MME is a noteworthy finding. Anecdotally, clinicians frequently assume that patients using COAT with a higher MME are more likely to be refractory to cessation. As a result of this, so many patients have been ostracized from medical clinics based on their high MME values that popular media has coined the term “Opioid Refugee” [94] to describe their inability to find clinicians to maintain their prescriptions. A better understanding of the anxiety and fear-based factors that contribute to COAT reliance would enable more a more pragmatic approach to clinical problem identification and targeted treatment, which could help improve healthcare quality and accessibility by reincorporating these highly exposed patients into the medical system.

It is the goal of the researchers that the data presented here may inform the effort within the medical field toward continuing to establish efficient and effective best-practice approaches for the treatment of patients with CNCP who rely upon COAT. It has been documented that targeted educational campaigns matching specific treatment to certain patient characteristics can have a positive effect on beliefs and clinical outcomes [76, 80, 95, 96]. Specifically, studies have utilized trends in psychological assessments related to anxiety and fear-based beliefs and behavior to affect positive change in disability related to CNCP [61, 80]. One study found that successfully lowering fear avoidance scores in patients with chronic back pain, through an educational campaign, resulted in subsequently decreased reports of disability in the same patients, despite no improvement in pain [80]. Similarly, a curriculum designed to confront pain catastrophizing and fear of reinjury, coupled with physical therapy, had a 25% higher return to work rate than physical therapy alone [61]. The current findings, when coupled with these previously documented clinical applications, suggest that there could be efficacy in a model of targeted mind-based assessment and treatment to confront anxiety and fear-based beliefs and behavior in the effort to reduce refractory COAT reliance.

The data above are presented as preliminary due to the fact that it was collected in such a way as to make it vulnerable to factors that dilute generalizability. The data was gathered retrospectively from a modestly sized and specific group of nonrandomized, nonblinded patients who were treated in a private practice setting. The subjects were selected to knowingly participate in patient care-planning toward an agreed upon goal of COAT cessation, which is a design weakness, despite the fact that the inclusion criteria required prior attempts and failures at opioid weaning and cessation, which suggests that this population was actually at higher risk for refractory reliance. Further, though varied insurance payer sources suggest a variety of socioeconomic backgrounds among participants, demographic data are limited. However, studies showing focused potential targets for interventions to decrease reliance upon COAT are valuable and scarce. Thus, we argue that sharing information gathered from such a notable clinical observation—as was seen here—is contributory, despite the limitations inherent in the study design.

## Conclusion

The present results suggest that fear avoidance beliefs and behavior, as measured by the FAB-PA, play a significant role in refractory COAT reliance for patients with CNCP and that incoming MME and COMM scores are noncontributory. While reproducibility of these preliminary results in larger and more varied settings will be key to better understanding the relationship between COAT cessation and anxiety and fear-based beliefs and behavior, this study builds upon an on-going discussion within the medical community aiming to identify and address factors relating to refractory COAT reliance. The investigators are hopeful that insights gained from this report will help researchers and clinicians narrow the scope of inquiry around useful assessments and interventions for best-practice approaches to COAT cessation in the population with CNCP.

## Notes

Disclosure: The Primary Investigator of this research was the Founder and Medical Director of the multidisciplinary clinical program described. The program is no longer in session, and there is no financial incentive tied to the inadvertent publicity this potential publication could provide. The researchers analyzed the data presented here through the course of program-related quality assurance measures. No other funding was present for this study. This study was reviewed by a private IRB and was determined exempt from full review.
